# Supplementary material for: Tissue-Specific Evolution of Protein Coding Genes in Human and Mouse
Source: PLoS One. 2015 Jun 29;10(6):e0131673. doi: 10.1371/journal.pone.0131673 (PMC4488272; doi:10.1371/journal.pone.0131673)
Supplement: S1 File — (PDF) [file pone.0131673.s001.pdf]

Supplementary Material 1

Parameters used for analysis:

Negative selection (Omega, Omega 0, ω_0), neutral evolution (p_1), positive selection (ΔlnL), tissue specificity (tau, τ), median expression, maximal expression, stage number, GC content, intron length, intron number, CDS length, paralogs number, phyletic age.

Supplementary Material online:

Can be downloaded from: http://dx.doi.org/10.6084/m9.figshare.1221771

Files 1 – 6: Data used for analysis for the 6 different data sets.

Files 7 – 30: Results of correlation analysis.

Files 31 – 36: Data used for analysis with expression for each tissue separately.

Files 37 – 60: Results of correlation analysis for each tissue separately.

Files 61 – 66: Results of correlation analysis for tissue specific genes ($\tau > 0.2$).

Files 67 – 72: Results of correlation analysis for each tissue separately for tissue specific genes ($\tau > 0.2$).

Files 73 – 78: Results of correlation analysis for genes without available evolutionary data.

File 79: Results of correlation analysis with MI score instead of omega 0.

Files 80 – 81: Results of correlation analysis with mean expression, instead of median expression.

Files 82 – 83: Data used for analysis with most expressed transcript instead of the longest transcript.

Files 84 – 85: Results of correlation analysis with most expressed transcript instead of the longest transcript.

Files: 86 – 87: Data and results of correlation analysis with recombination rate and connectivity.

Figure 1 – 24: Results of correlation analysis.

Figures 25 – 48: Results of correlation analysis for each tissue separately.

Figures 49 – 54: Results of correlation analysis for tissue specific genes ($\tau > 0.2$).

Figures 55 – 60: Results of correlation analysis for each tissue separately for tissue specific genes ($\tau > 0.2$).

Figures 61 – 66: Results of correlation analysis for genes without available evolutionary data.

Figures 67 – 68: Results of correlation analysis with mean expression, instead of median expression.

Figures 69 – 70: Results of correlation analysis with most expressed transcript instead of the longest transcript.

Figure 71: Results of correlation analysis with recombination rate and connectivity.

Key for file names:

Hum = Human; Mus = Mouse

Partial = Partial correlations results; Normal = Standard correlations results

Spearman = Spearman correlations; Pearson = Pearson correlations

Tissues = data for tissues separately

Fagerberg = (Fagerberg et al. 2013); Bgee = microarray Bgee data base (Bastian et al. 2008); Brawand = (Brawand et al. 2011)

Supplementary table A: Example of calculation of partial correlation. The data used are the height of the person (random numbers between 160cm and 190cm) and length of both legs (randomly smaller 1 to 3 cm).

Data$High <- runif(10 000, 160, 190)<br>Data$LeftLeg <- data$High -100 –runif(10000,1,3)<br>Data$RightLeg <- data$High – 100 – runif(10000, 1, 3)		
	Simple correlation	Partial correlation
high vs. left leg:	R = 0.9977	R = 0.7089
high vs. right leg:	R = 0.9977	R = 0.7031
left leg vs. right leg	R = 0.9955	R = 0.0008
Data$RightLeg <- data$LeftLeg – runif(10000, 1, 3)		
high vs. left leg:	R = 0.9977	R = 0.7023
high vs. right leg:	R = 0.9956	R = 0.0042
left leg vs. right leg	R = 0.9978	R = 0.7072

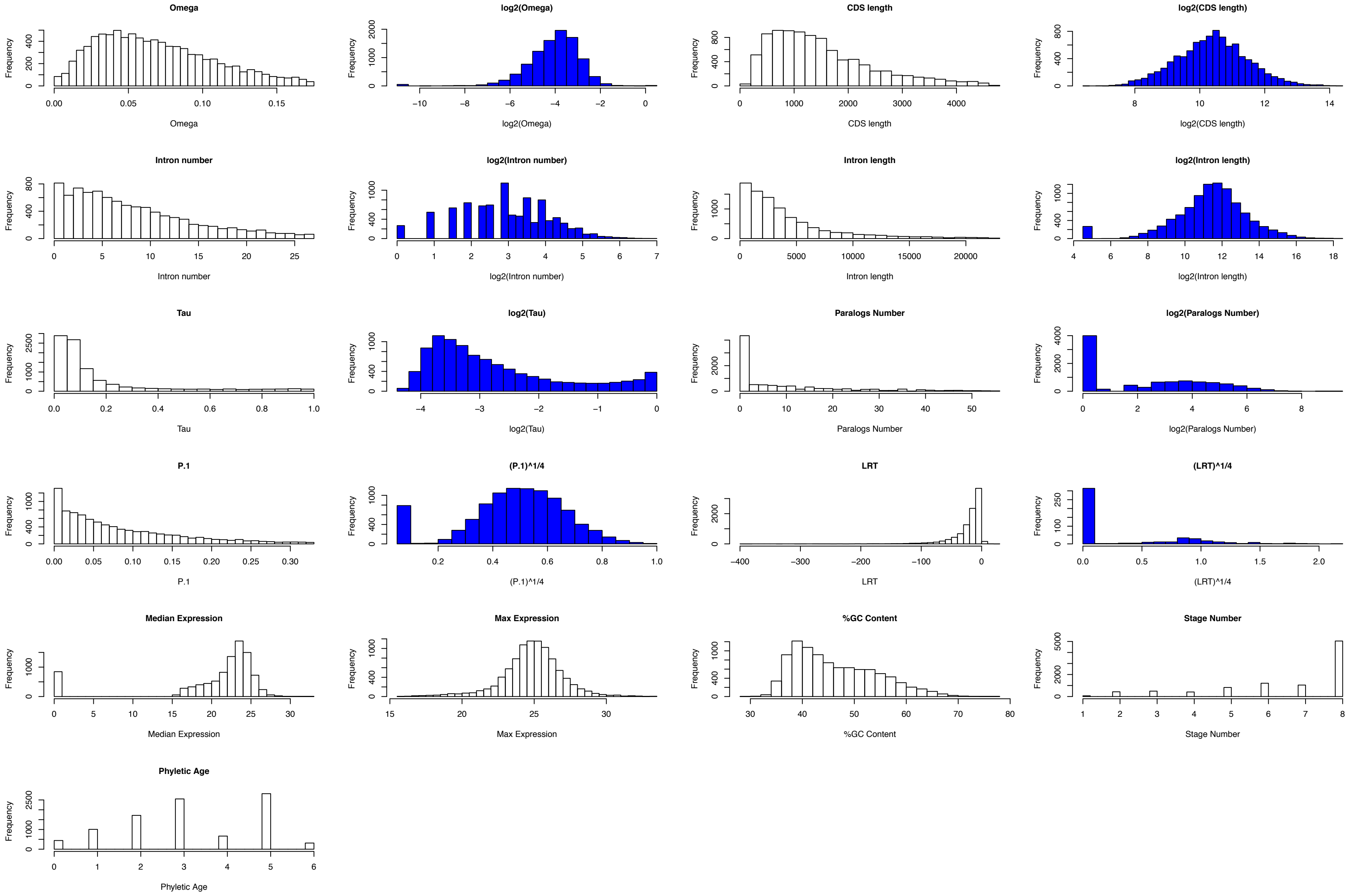

Supplementary figure A: Distribution of all parameter values for human data with expression values from Fagerberg et al (Fagerberg et al. 2013).

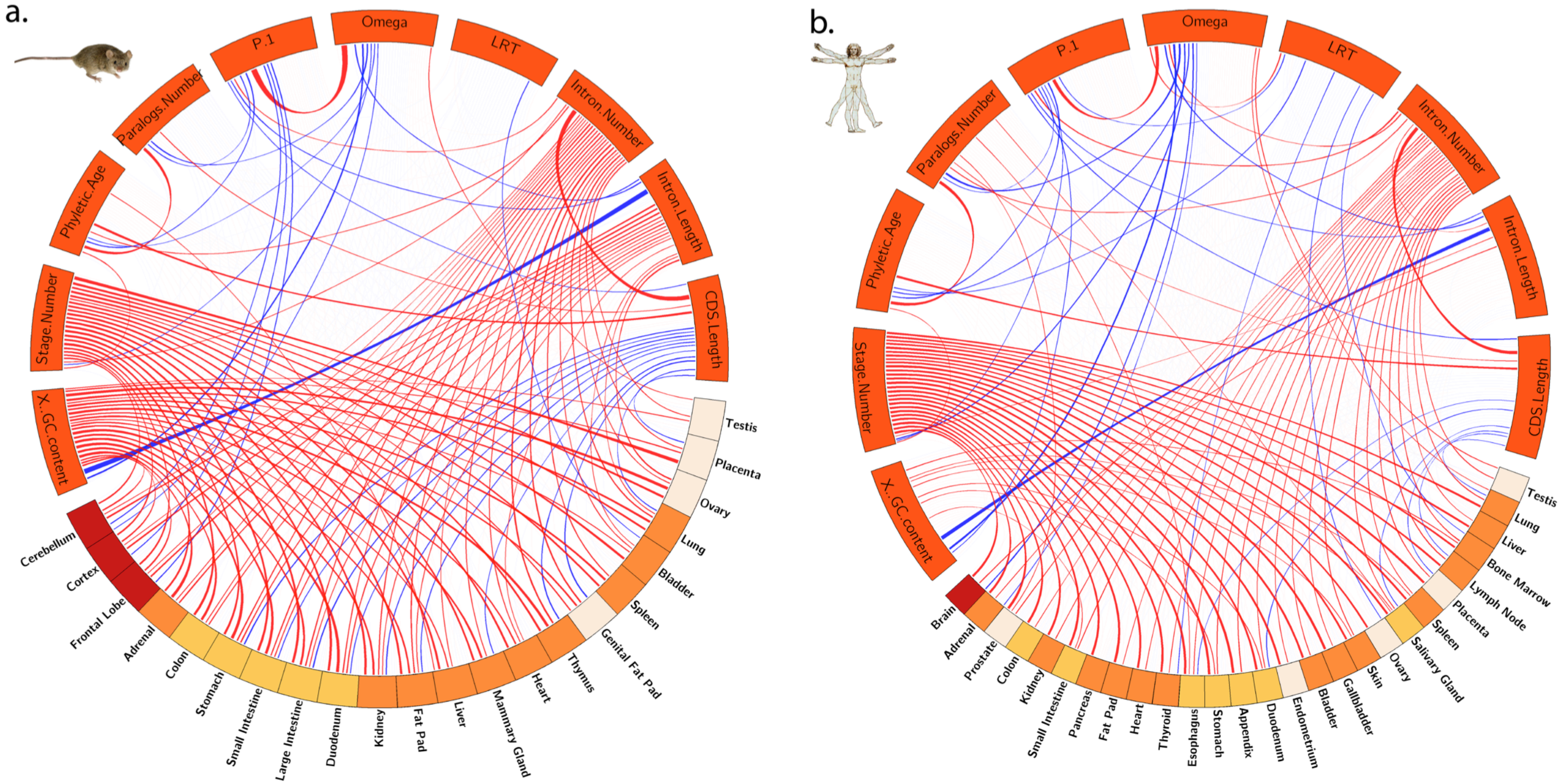

Supplementary figure B: Spearman partial correlation with expression values (only tissue specific genes, $\tau > 0.2$) for each tissue separately for a) mouse and b) human. The width of the lines shows the strength of correlations. Red lines show positive correlations, blue shows negative correlations. Only significant correlations ($p<0.0005$) are shown. Color of the bands represents different groups of tissues (gastrointestinal system, central nervous system, reproductive system and divers).

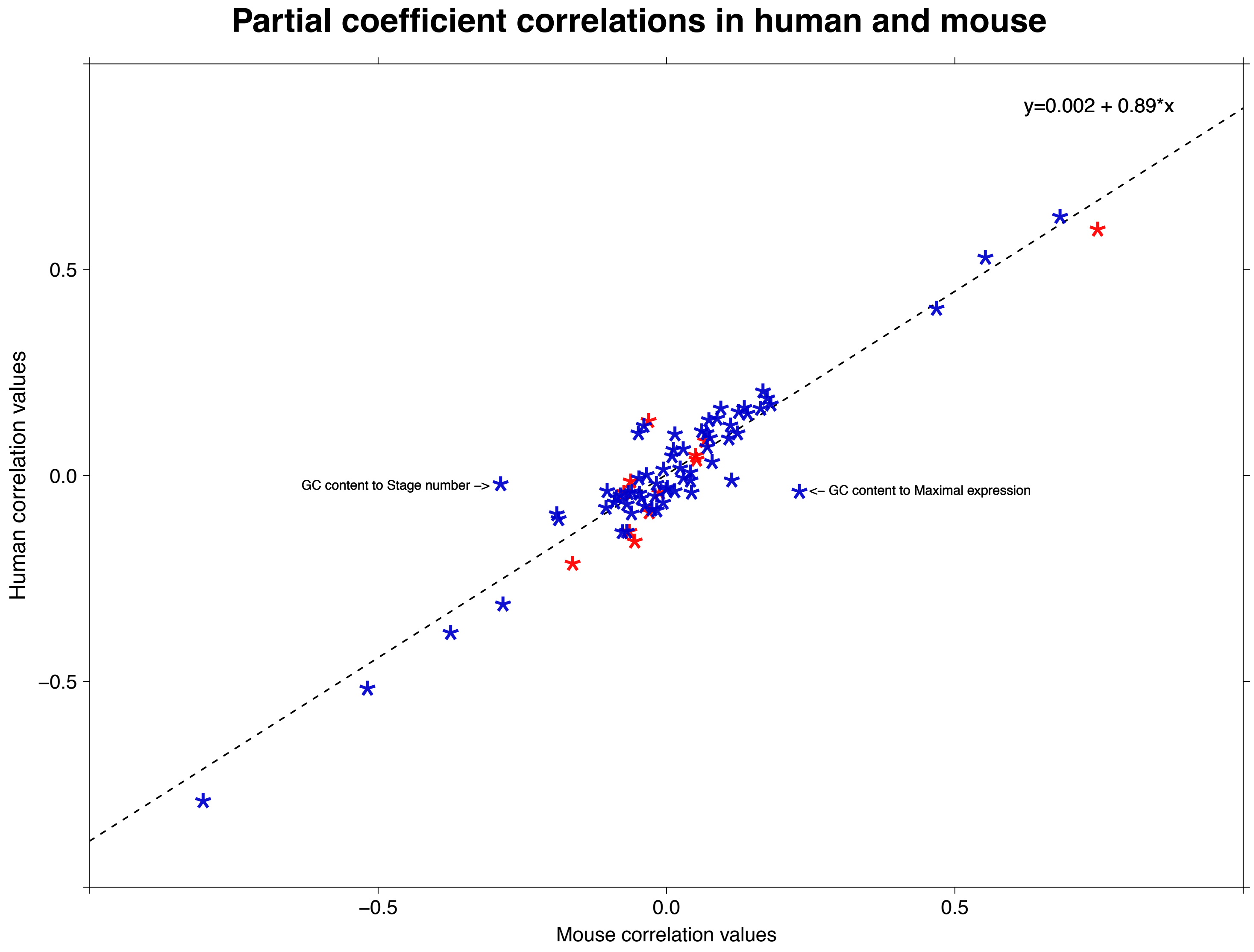

Supplementary figure C: Comparison of Spearman partial correlation coefficients between human and mouse. In red, correlations involving ω_0.

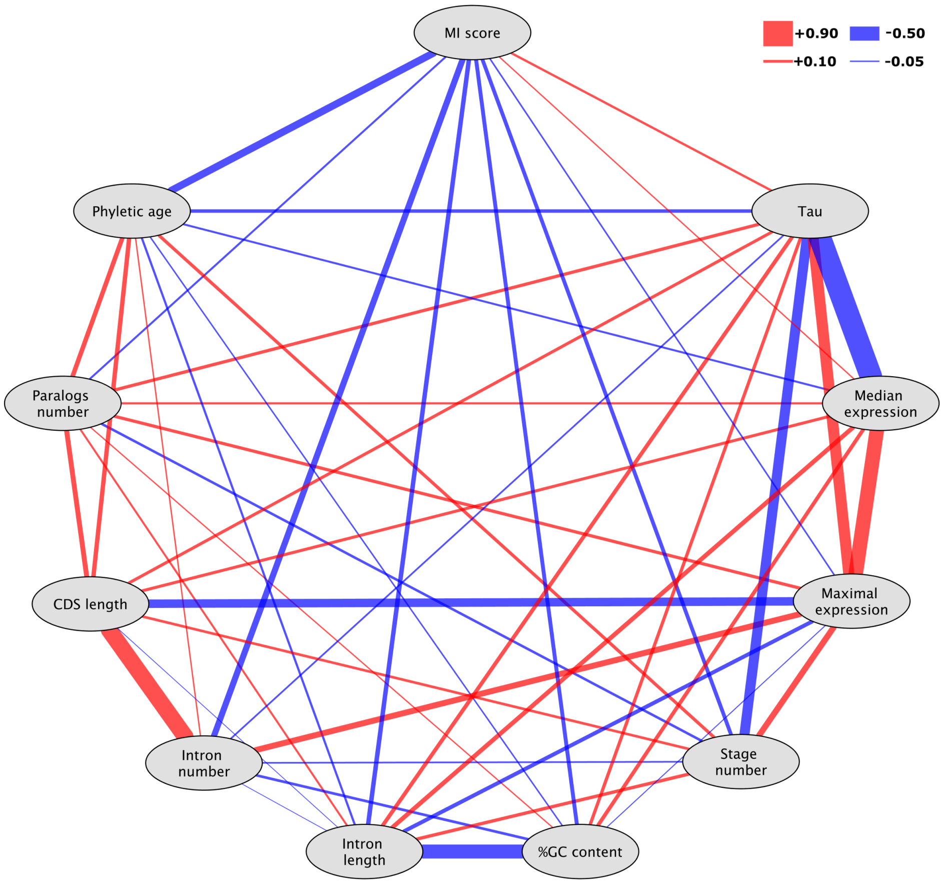

Supplementary figure D: Spearman partial correlations human using MI score to represent evolutionary rate. The width of the lines shows the strength of correlations. Red lines show positive correlations, blue shows negative correlations. Only significant correlations (p<0.0005) are shown.

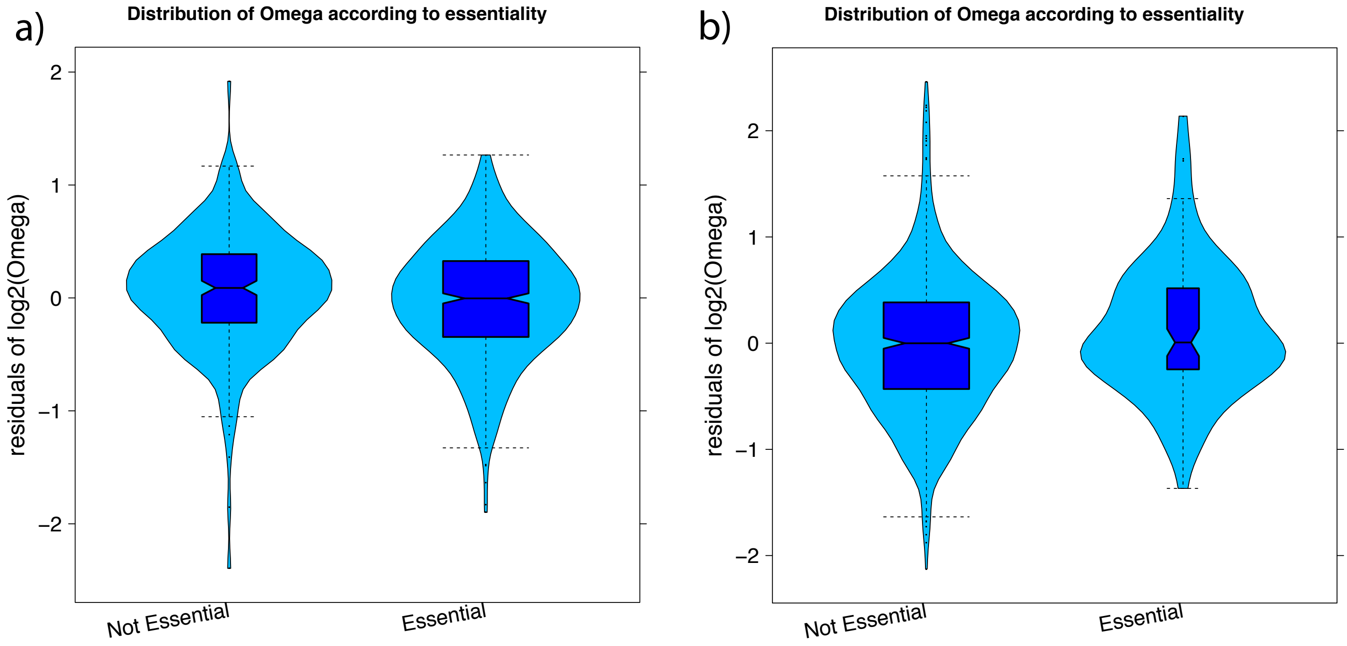

Supplementary figure E: Distribution of Omega 0 residuals according to essentiality in a) mouse (t-test p = 0.03041) and b) human (t-test p = 0.05607).

Example of command line for running analysis for ENCODE data, as described in Material and Methods:

tophat -p 4 —-no-novel-juncs —-no-novel-indels -G Mouse_gtf_file.gtf —-b2-very-fast —-transcriptome-index=Mouse.index -x 1 -T -o Mouse_organ_output Mouse_genome Organ_reads.fastq

cufflinks -p 4 -G Mouse_gtf_file -o Mouse_organ_output Mouse_organ_output.bam

References

Bastian F, Parmentier G, Roux J. 2008. Bgee: integrating and comparing heterogeneous transcriptome data among species. Data Integr. … [Internet]:124–131. Available from: http://link.springer.com/chapter/10.1007/978-3-540-69828-9_12

Brawand D, Soumillon M, Necsulea A, et al. 2011. The evolution of gene expression levels in mammalian organs. Nature [Internet] 478:343–348. Available from: http://www.ncbi.nlm.nih.gov/pubmed/22012392

Fagerberg L, Hallstrom BM, Oksvold P, et al. 2013. Analysis of the human tissue-specific expression by genome-wide integration of transcriptomics and antibody-based proteomics. Mol. Cell. Proteomics [Internet]. Available from: http://www.ncbi.nlm.nih.gov/pubmed/24309898

The ENCODE Project Consortium. 2007. Identification and analysis of functional elements in 1% of the human genome by the ENCODE pilot project. Nature [Internet] 447:799–816. Available from: http://www.pubmedcentral.nih.gov/articlerender.fcgi?artid=2212820&tool=pmcentrez&rendertype=abstract
